# Electronic Screening for Alcohol Use and Brief Intervention by Email for University Students: Reanalysis of Findings From a Randomized Controlled Trial Using a Bayesian Framework

**DOI:** 10.2196/14419

**Published:** 2019-11-07

**Authors:** Marcus Bendtsen

**Affiliations:** 1 Department of Medical and Health Sciences Linköping University Linköping Sweden

**Keywords:** Bayesian analysis, telemedicine, alcohol, randomized controlled trial

## Abstract

**Background:**

Almost a decade ago, Sweden became the first country to implement a national system enabling student health care centers across all universities to routinely administer (via email) an electronic alcohol screening and brief intervention to their students. The Alcohol email assessment and feedback study dismantling effectiveness for university students (AMADEUS-1) trial aimed to assess the effect of the student health care centers’ routine practices by exploiting the lack of any standard timing for the email invitation and by masking trial participation from students. The original analyses adopted the conventional null hypothesis framework, and the results were consistently in the expected direction. However, since for some tests the *P* values did not pass the conventional .05 threshold, some of the analyses were necessarily inconclusive.

**Objective:**

The outcomes of the AMADEUS-1 trial were derived from the first 3 items of the Alcohol Use Disorders Identification Test (AUDIT-C). The aim of this paper was to reanalyze the two primary outcomes of the AMADEUS-1 trial (AUDIT-C scores and prevalence of risky drinking), using the same models used in the original publication but applying a Bayesian inference framework and interpretation.

**Methods:**

The same regression models used in the original analysis were employed in this reanalysis (linear and logistic regression). Model parameters were given uniform priors. Markov chain Monte Carlo was used for Bayesian inference, and posterior probabilities were calculated for prespecified thresholds of interest.

**Results:**

Where the null hypothesis tests showed inconclusive results, the Bayesian analysis showed that offering an intervention at baseline was preferable compared to offering nothing. At follow-up, the probability of a lower AUDIT-C score among those who had been offered an intervention at baseline was greater than 95%, as was the case when comparing the prevalence of risky drinking.

**Conclusions:**

The Bayesian analysis allows for a more consistent perspective of the data collected in the trial, since dichotomization of evidence is not looked for at some arbitrary threshold. Results are presented that represent the data collected in the trial rather than trying to make conclusions about the existence of a population effect. Thus, policy makers can think about the value of keeping the national system without having to navigate the treacherous landscape of statistical significance.

**Trial Registration:**

ISRCTN Registry ISRCTN28328154; http://www.isrctn.com/ISRCTN28328154

## Introduction

### Background

Alcohol consumption contributed to more than 4.5% of deaths globally in 2016 [1] and was the leading risk factor among the population aged 15-49 years old. It has been suggested that alcohol policies might need to be revised worldwide to lower the overall population-level consumption [2]. While policies controlling price and availability may be one way forward [3], the advent of electronic health (eHealth) interventions has made us better equipped to deliver personal behavior change interventions to larger populations.

Early initiatives to use digital means of delivering alcohol interventions came in the form of electronic screening and brief interventions (eSBIs) [4-8]. Typically, these interventions ask participants to complete a questionnaire, after which feedback is given on their responses and some advice on behavior change is offered (based on recommended drinking levels). The feedback and advice are commonly designed around behavior change theories and models, such as protection motivation theory [9], social cognitive theory [10] and the theory of planned behavior [11]. In general, eHealth interventions for alcohol behavior change have shown promise when they have included components that focus on behavior substitution, problem solving, goal setting, review of behavioral goals, self-monitoring, and normative feedback [12,13].

Meta-analyses suggest that there exists a small positive effect of eSBIs on the amount of alcohol consumed weekly in the short term, with a Cohen *d*=−0.17 (95% CI −0.27 to −0.18) found in one analysis [14], a Cohen *d*=−0.14 (95% CI −0.24 to −0.03) in another analysis [15], and a weighted mean difference of alcohol in grams=−16.59 (95% CI −23.70 to −9.48) in a third analysis [16]. Although long-term effects have not been measurable, these brief interventions are nevertheless useful for reaching many individuals at a low cost.

Almost a decade ago, Sweden became the first country to implement a national system enabling student health care centers across all universities to routinely administer eSBIs. The system, which is still routinely used today, sends an email to all university students with an invitation and a hyperlink to a 10-item web questionnaire which is then followed by personal feedback and advice. At the time this system was introduced, there was some evidence of the effectiveness of eSBIs but there was a paucity for large-scale, multisite, effectiveness trials of routine care systems.

### The Alcohol Email Assessment and Feedback Study Dismantling Effectiveness for University Students Trial

The Alcohol email assessment and feedback study dismantling effectiveness for university students (AMADEUS-1) trial [4,7], conducted in 2011, aimed to assess the effect of the student health care centers’ routine practices by exploiting the lack of any standard timing of the email invitation and by masking trial participation from students. The trial outcome was originally reported in 2013 [7].

During the autumn term of 2011, all students in semesters 1, 3 and 5 at two universities in Sweden (Linköping and Luleå) were included in the AMADEUS-1 trial. Notably, students’ email addresses were randomized into 3 groups (Group 1, Group 2, and Group 3) prior to any invitation or contact with the students. Ethical concerns with the use of this type of masking was considered and approved by the Regional Ethical Committee in Linköping, Sweden (No 2010/291-31). In a subsequent trial (the AMADEUS-2 trial [6,8]) a more conventional approach was used to estimate the effect of eSBIs on harmful and hazardous drinkers. A Bayesian reanalysis of the AMADEUS-2 trial has also been reported [17].

On September 5, 2011, Group 1 and Group 2 were sent an email from the student health care center with a hyperlink to a web questionnaire comprising 10 items which assessed their current alcohol consumption, masked as part of routine care. Group 1 was additionally told that they would also get feedback, which they received immediately after responding to the questionnaire. Group 2 was thanked for their participation and offered a hyperlink to a website with general information about alcohol, which was not believed to have any content helpful for supporting behavior change. Group 3 was not contacted at this time.

Three months after the initial email to Group 1 and Group 2, all three groups were sent identical emails with an invitation to participate in a web-based general lifestyle survey where 3 out of the 15 items were the first 3 items of the Alcohol Use Disorders Identification Test (AUDIT-C [18]). Crucially, this invitation made no reference to the alcohol assessment conducted three months earlier and it was not disclosed as a follow-up questionnaire in a randomized trial.

### Objectives

Outcomes of the AMADEUS-1 trial were derived from the 3 AUDIT-C items in the general lifestyle survey. This reanalysis will focus on two primary outcomes: AUDIT-C scores and prevalence of risky drinking. In Sweden, risky drinkers are those who fulfil at least one of two criteria: (1) heavy episodic drinking of at least 4 (female) or 5 (male) standard drinks of alcohol on one occasion the past month; or (2) consuming more than 9 (female) or 14 (male) standard drinks of alcohol per week. One standard drink is defined as 12 grams of alcohol in Sweden.

The current goal is to reanalyze the two primary outcomes of the AMADEUS-1 trial, using the same models used in the original publication but also using a Bayesian inference framework and interpretation.

## Methods

### Overview

In the original analysis of the AMADEUS-1 trial, normal regression was used to contrast AUDIT-C scores (log-transformed) and logistic regression was used to contrast risky drinking. Both models were adjusted for baseline variables. In this Bayesian analysis, the same regression models were used, and uniform priors were applied to all model parameters. The full specifications of the Bayesian models can be seen in the following 2 equations. Separate analyses were done comparing Group 1 versus Group 3 and Group 2 versus Group 3. In all cases, Group 3 was considered the control group and Group 1 and Group 2 were considered intervention groups.

Equation 1:

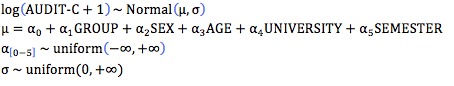



Equation 2:

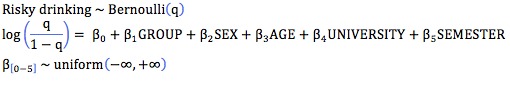



### Alcohol Use Disorders Identification Test

When contrasting AUDIT-C scores (Equation 1), the primary interest of the analyses was the regression coefficient for the group variable (α_1_). A negative value for α_1_ suggests that the group which was randomized to receive an intervention (Group 1 and Group 2 respectively) had, on average, lower AUDIT-C scores at follow-up than the group which was randomized to the control setting (Group 3). Coefficients were back transformed prior to inspection. Informed by the original analysis, it was decided that thresholds of interest for which the marginal posterior distribution for α_1_ should be inspected were 0, –0.02, and –0.04. The thresholds were chosen to communicate whether offering an intervention is preferable to not doing so (the 0 threshold), and to indicate the magnitude of the difference between groups (–0.02 and –0.04).

### Risky Drinking

When contrasting risky drinking (Equation 2), the primary interest was the regression coefficient for the group variable (β_1_), that is the log of the odds ratio (OR) between the group which was randomized to an intervention (Group 1 and Group 2 respectively) and the group which was randomized to the control setting (Group 3). Coefficients were exponentiated before inspection, thus a value of β_1_ lower than 1 would suggest that the odds of risky drinking in the intervention group was lower than the odds in the control. Informed by the original analysis, it was decided that thresholds of interest for which the marginal posterior distribution for β_1_ should be inspected was 1, 0.9 and 0.8. Again, the thresholds were chosen to communicate whether offering an intervention is preferable to not doing so (the 1 threshold), and to indicate the magnitude of the difference between the groups (0.9 and 0.8).

### Inference

Markov chain Monte Carlo was used for Bayesian inference (RStan version 2.16.2). For each model, 50,000 iterations were run with 25,000 warmup iterations in four chains. Inference for AUDIT-C scores (Equation 1) took 3.5 minutes, and for risky drinking (Equation 2) 5.5 minutes. All computations were done on a MacBook Pro (2017 model).

## Results

### Primary Findings

A total of 14,910 students were randomized into the 3 arms of the trial. In Group 1, 36.2% (1798/4969) of participants completed the eSBI, 32.6% (1621/4969) of Group 2 participants completed the alcohol screening questionnaire, and as previously discussed Group 3 was not contacted at this point. Approximately half of all students responded to the general lifestyle survey that was sent three months after randomization: 51.2% (2546/4969) in Group 1, 52.2% (2594/4969) in Group 2, and 53.7% (2669/4972) in Group 3.

### Original Analysis: Null Hypothesis Framework

The original analysis for the AMADEUS-1 trial is presented in Table 1 [7]. Null hypothesis tests were two-tailed and assessed at the .05 threshold. It was found that Group 1 and Group 3 did not report a statistically significant difference with respect to AUDIT-C scores (*P*=.07) while Group 2 and Group 3 did (*P*=.04), with Group 2, on average, reporting a lower AUDIT-C score than Group 3. Risky drinking was found to be statistically significantly different between Group 1 and Group 3 (*P*=.006), with risky drinking less prevalent in Group 1 than in Group 3, but not so for Group 2 and Group 3 (*P*=.08).

As a reminder, *P* values indicate how likely it is that we would have seen the data that we did in the trial in a hypothetical world where the population effect is exactly zero. Convention says that if the data is less likely than 5%, then we should reject the hypothetical world. However, it does not mean that if the 5% threshold is not broken that we should accept the hypothetical world. Instead, the confidence interval indicates which hypothetical worlds cannot be rejected given the data that we have seen in the trial.

**Table 1:** Original analysis of AUDIT-C and risky drinking at follow-up, comparing Group 1 versus 2 and Group 2 versus 3.

Categories	Group 1 (n=2546)	Group 2 (n=2594)	Group 3 (n=2669)	Group 1 versus 3		Group 2 versus 3	
				Regression coefficient^a^, 95% CI	*P* value	Regression coefficient^a^, 95% CI	*P* value
AUDIT-C^b^	3.46 (3.09)^c^	3.44 (3.17)^c^	3.60 (3.14)^c^	–0.032 (–0.066 to 0.003)	.07	–0.038 (–0.072 to –0.002)	.04
Risky drinking^d^	1136 (44.6)^e^	1194 (46.0)^e^	1288 (48.3)^e^	0.85 (0.76 to 0.95)	.006	0.90 (0.81 to 1.01)	.08

^a^Linear coefficient for AUDIT-C scores (back transformed) and odds ratio for risky drinking (adjusted for sex, age, university, and semester).

^b^AUDIT-C: Alcohol Use Disorders Identification Test.

^c^Geometric mean (SD). Approximate standard deviation back-calculated from the log-scale.

^d^Risky drinking: heavy episodic drinking ≥1 a month or weekly consumption >14 for men and >9 for women (Swedish national guidelines).

^e^n (%).

### Bayesian Analysis

**Figure 1:**
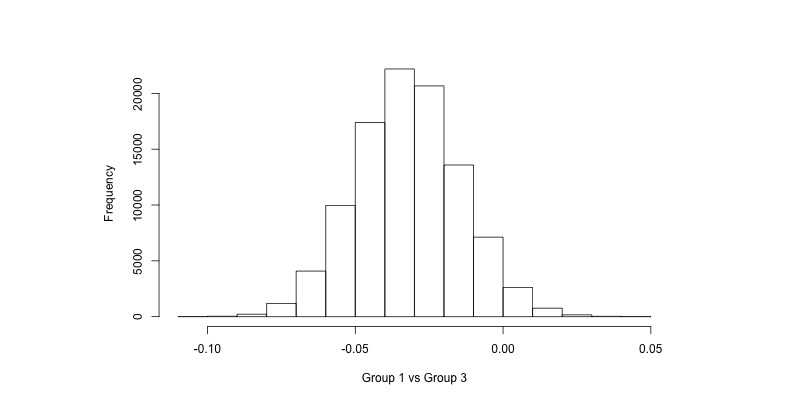
Samples from the posterior distribution of α_1_ in the AUDIT-C model when comparing Group 1 versus Group 3 (Equation 1, back transformed). AUDIT-C: Alcohol Use Disorders Identification Test.

**Figure 2:**
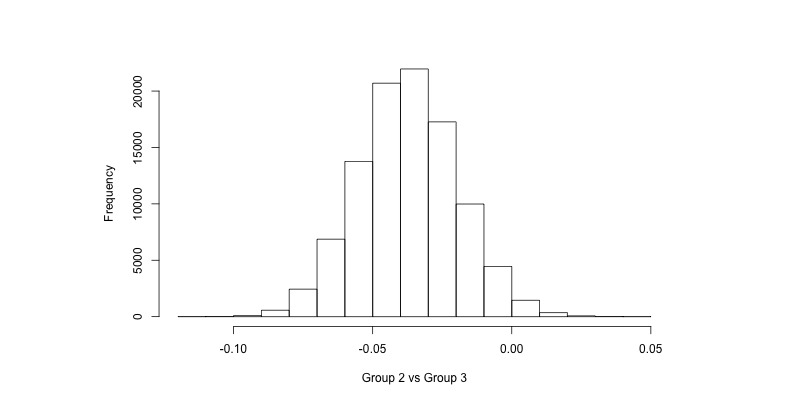
Samples from the posterior distribution of α_1_ in the AUDIT-C model when comparing Group 2 versus Group 3 (Equation 1, back transformed). AUDIT-C: Alcohol Use Disorders Identification Test.

**Figure 3:**
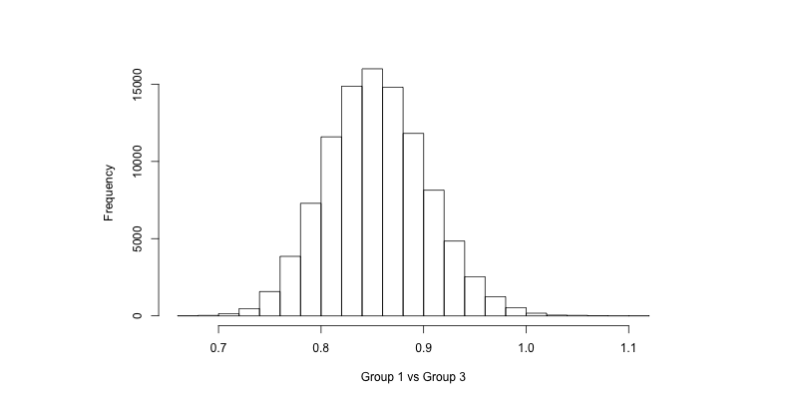
Samples from the posterior distribution of β_1_ in the risky drinking model when comparing Group 1 versus Group 3 (Equation 2, exponentiated).

**Figure 4:**
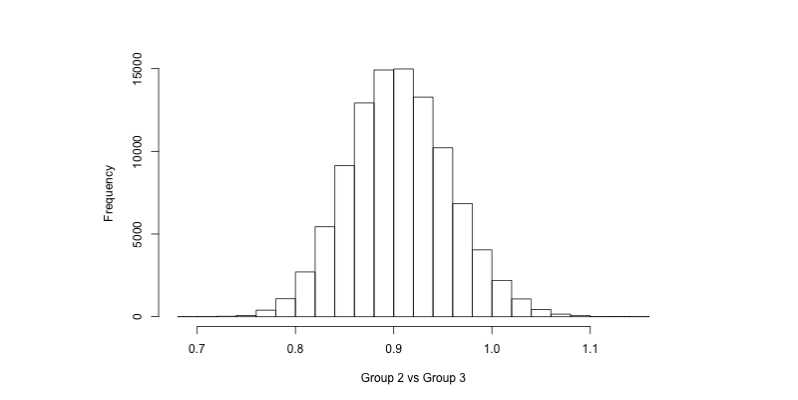
Samples from the posterior distribution of β_1_ in the risky drinking model when comparing Group 2 versus Group 3 (Equation 2, exponentiated).

The computational result of a Bayesian analysis using Markov chain Monte Carlo uses samples from the posterior distribution of each parameter of interest. Histograms of these samples are shown in Figures 1-4 for the coefficient for the group variable in the AUDIT-C models (α_1_ in Equation 1, back transformed) and the risky drinking models (β_1_ in Equation 2, exponentiated). For instance, in Figure 1 we can see that there is a majority of samples to the left of 0, indicating that it is more likely than not that there was a difference in AUDIT-C scores at follow-up between Groups 1 and 3. Similarly, in Figure 4 we can see that a majority of the samples are to the left of 1, indicating that the prevalence of risky drinking in Group 2 was lower than in Group 3 at follow-up (ie, the OR was lower than 1). For the enclosed analyses, no trends were found in the sampling when inspecting trace plots (see Multimedia Appendix 1).

Rather than just visually inspecting the histograms, the samples drawn during inference can be used to calculate probabilities of interest (Tables 2 and 3). For example, when comparing the prevalence of risky drinking between Group 1 and Group 3 in Table 3, the ratio of samples that were lower than 1 was 99.7%, thus there was a 99.7% probability that the OR was less than 1 (indicating fewer risky drinkers in Group 1 compared to Group 3). Furthermore, there was an 82.4% probability that the OR was less than 0.9.

**Table 2:** Bayesian analysis of AUDIT-C at follow-up comparing Group 1 versus 3 and Group 2 versus 3.

	Group 1 versus 3			Group 2 versus 3		
	Threshold 1	Threshold 2	Threshold 3	Threshold 1	Threshold 2	Threshold 3
Regression coefficient^a^ (AUDIT-C^b^)	<0	<–0.02	<–0.04	<0	<–0.02	<–0.04
Marginal posterior probability (%)	96.4	75.7	32.9	98.1	83.7	44.4

^a^Back transformed linear regression coefficient (model adjusted for sex, age, university, and semester).

^b^AUDIT-C: Alcohol Use Disorders Identification Test.

**Table 3:** Bayesian analysis of risky drinking at follow-up comparing Group 1 versus 3 and Group 2 versus 3.

	Group 1 versus 3			Group 2 versus 3		
	Threshold 1	Threshold 2	Threshold 3	Threshold 1	Threshold 2	Threshold 3
Odds ratio^a^ (Risky drinking)	<1	<0.9	<0.8	<1	<0.9	<0.8
Marginal posterior probability (%)	99.7	82.4	13.4	96.1	46.7	1.6

^a^Logistic regression coefficient in terms of odds ratios (model adjusted for sex, age, university, and semester).

## Discussion

### Key Findings

When comparing the analysis done in a null hypothesis framework with one done within the Bayesian framework, it is important to remind oneself of what the quantities represent as the questions being asked and answered are different.

The null hypothesis testing approach aims to put forth evidence about the population value of a parameter (ie, the existence of an effect on the entire population). The *P* value indicates how extreme the collected data are, given a fixed population value which is often set at a no-effect level. If the data is unlikely to have been generated from a population where the intervention has no effect, then the null hypothesis is rejected, and we can state that, with statistical significance, we believe that the intervention has a population effect. The fundamental issues with this approach have been discussed elsewhere [19-26], as has the problematic misinterpretation of *P* values and confidence intervals [27,28].

On the other hand, the Bayesian approach only concerns itself with the data at hand. It does not attempt to say anything about a population level effect, but instead calculates posterior distributions over model parameters. We can use these posterior distributions to calculate the probability of there being a difference between groups with respect to different trial outcomes.

### Alcohol Use Disorders Identification Test

#### Null Hypothesis Framework

When contrasting AUDIT-C scores (Equation 1) using the null hypothesis framework (Table 1) we found no significant difference between Group 1 and Group 3 (*P*=.07), but there was a significant difference between Group 2 and Group 3 (*P*=.04). This is somewhat counterintuitive, as Group 1 and 2 were given identical questionnaires, but Group 1 was also given feedback and advice. However, due to the nature of null hypothesis testing, we cannot discuss the effect of the feedback and advice component, since the very existence of an effect cannot be determined. Yet, we are to conclude that there does exist an effect with respect to responding to the questionnaire itself. Both *P* values are close to the conventional threshold of .05, and it is noteworthy that the entire discussion about effects would have changed had we adopted a .08 threshold or a .03 threshold.

In the original report [7], a direct comparison between Group 1 and Group 2 was also included but has been left out of this reanalysis for succinctness. The results were inconclusive, with no *P* values crossing the conventional threshold.

#### Bayesian Framework

The Bayesian approach (Table 2) suggests that the probability that Group 1 had a lower AUDIT-C score on average compared to Group 2 at follow-up was 96.4%, and when comparing Group 2 and Group 3 this probability was 98.1%. The model does not make a dichotomous decision about the existence of an effect but states the probability that a difference existed between the groups. Licensed by the randomization component of the trial design, we may conclude that this difference is due to the groups receiving different treatments. We may also conclude that the difference between groups is more likely than not to be greater than 0.02 units on the AUDIT-C scale but also is more likely than not to be less than 0.04.

### Risky Drinking

#### Null Hypothesis Framework

In Table 1 we can see that the difference in prevalence of risky drinking between Group 1 and Group 3 was statistically significant, but not between Group 2 and Group 3. The existence of an effect on risky drinking is thus confirmed for the questionnaire plus feedback and advice intervention, but the evidence is inconclusive for the effect of the questionnaire alone. Recall that the situation was the opposite when analyzing AUDIT-C scores.

#### Bayesian Framework

The Bayesian approach (Table 3) suggests that there is a 99.7% probability that the prevalence of risky drinking was lower in Group 1 compared to Group 3, and that this probability was 96.1% when comparing Group 2 and Group 3. Note, however, that it was more likely than not that the OR was less than 0.9 when comparing Group 1 and Group 3, but not so when comparing Group 2 and Group 3. We can also see this when comparing Figure 3 and Figure 4, as most of the samples drawn when comparing Group 1 and Group 3 are to the left of 0.9, while they are centered around 0.9 when comparing Group 2 and Group 3. As was the case with AUDIT-C scores, we may attribute the difference between groups to the different treatments licensed by the trial design.

### Clinical Significance

Clinically significant effect sizes are not universal, as they depend on the context in which the intervention can be offered and must be decided upon given cost, alternative interventions, ethical and practical concerns, and so on. One of the benefits of using a Bayesian approach is that we have access to a posterior distribution over the parameters of our model, which allows us to answer questions such as, “What is the probability that the effect is X or greater?” Therefore, we can evaluate the probability of clinically significant effect sizes in several different contexts. For instance, at the time of the AMADEUS-1 trial, student health care centers in Sweden did not have any means of reaching the entire student population with a brief intervention, thus there were no alternative interventions to the eSBI on trial. In addition, there was very little cost involved in adopting the eSBI into routine practice. Tables 1 and 3 indicate that there was a 4-percentage point difference in risky drinking between Groups 1 and 3 (OR<0.9; 82.4% probability), and this was considered a significant enough effect size to mandate a full-scale adoption of the intervention.

The years to come after the AMADEUS-1 trial saw many more trials of eSBIs, and as was mentioned earlier, meta-analyses suggest a small positive effect of eSBIs on the amount of alcohol consumed weekly in the short term.

### Limitations

The AMADEUS-1 trial was unconventional in the sense that participants were randomized prior to being invited to the trial. This design allowed for a naturalistic study context and allowed for methodological advantages. However, participation rates were lower than would be expected in a more traditional setting where participants are randomized after registering interest in the trial (eg, only 36.2% [1798/4969] of participants allocated to Group 1 completed the eSBI). The overall follow-up rate was not remarkable at 52% (7764/14,910), which at the time was considered average for eHealth trials. Since missing at random cannot be guaranteed, effect sizes should be considered in the light that bias might have been introduced due to lower than ideal follow-up rates.

### Summary

In the original publication of the AMADEUS-1 trial, we summarized the main results as follows [7]: There were consistently small differences in the anticipated direction in comparisons with group 3, which were possibly as a result of chance, with *P*<.10 for four of five comparisons for both groups 1 and 2.

Since not all *P* values from the hypotheses tests passed conventional thresholds, we could not conclude that the different groups had been affected by treating them differently. Unfortunately, the desire to dichotomize evidence prohibited any further discussion. This dichotomization of evidence creates issues when interpreting results from trials, as clearly the contradicting results when contrasting AUDIT-C scores is difficult to explain and communicate. Thus, we may ask if it is prudent for the student health care centers to decide to change their policy of offering eSBIs to all students since the .05 threshold was not broken? What if a different threshold was chosen? Should the results simply be discarded as inconclusive?

In this Bayesian reanalysis, we may instead summarize our findings as: There is 96.4% probability that Group 1 had a lower AUDIT-C score on average than did Group 3, and there is a 99.7% probability that the prevalence of risky drinking was lower in Group 1 compared to Group 3 (and a further 82.4% probability that the OR was less than 0.9). This then allows us to go forth and inspect the posterior distributions at effect sizes that are clinically significant in different contexts and discuss whether the intervention should be adopted into routine practice.

## Appendix

Multimedia Appendix 1 Trace plots.

## Abbreviations

AMADEUS-1: Alcohol email assessment and feedback study dismantling effectiveness for university students
AUDIT-C: Alcohol Use Disorders Identification Test
eHealth: electronic health
eSBI: electronic screening and brief intervention
OR: odds ratio
