# Supplementary material for: Electronic Screening for Alcohol Use and Brief Intervention by Email for University Students: Reanalysis of Findings From a Randomized Controlled Trial Using a Bayesian Framework
Source: J Med Internet Res. 2019 Nov 7;21(11):e14419. doi: 10.2196/14419 (PMC6873145; doi:10.2196/14419)
Supplement: Multimedia Appendix 1 [file jmir_v21i11e14419_app1.pdf]

APPENDIX A – TRACE PLOTS

Trace plots can be useful to diagnose issues in sampling when using Monte Carlo methods. Figures 5 through 8 show trace plots for the four included analyses. Specifically, since there are no visible trends in the plots, the sampling was likely well dispersed over likely parameter values.

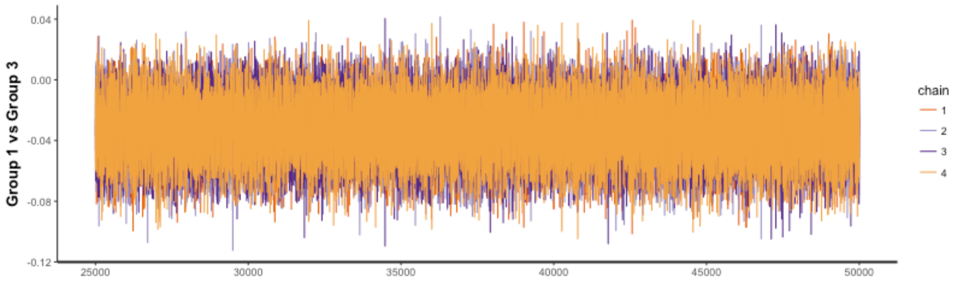

Figure 5 – Trace plot from Markov chain Monte Carlo sampling - AUDIT-C model (Equation 1) Group 1 vs 3

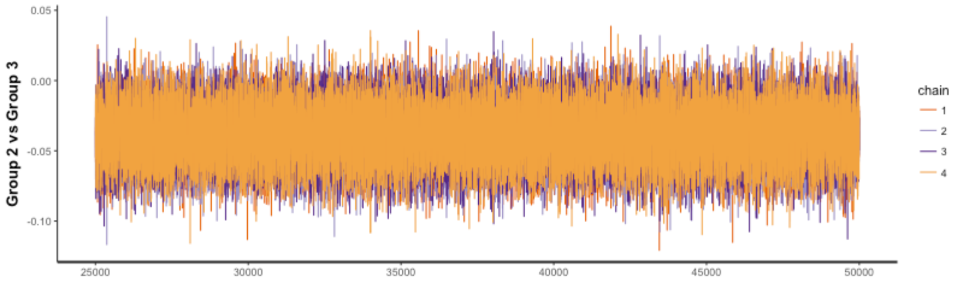

Figure 6 - Trace plot from Markov chain Monte Carlo sampling - AUDIT-C model (Equation 1) Group 2 vs 3

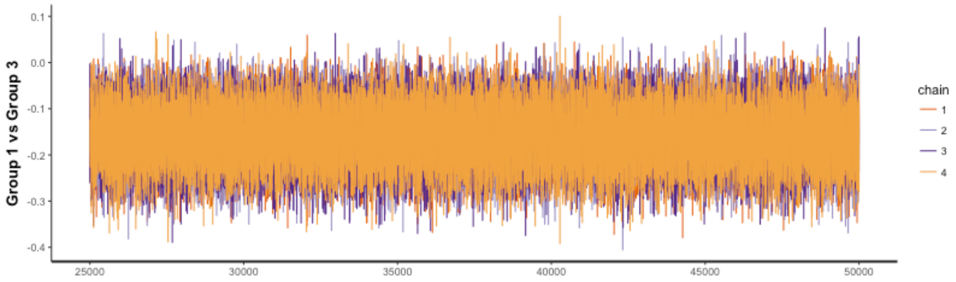

Figure 7 - Trace plot from Markov chain Monte Carlo sampling – risky drinking model (Equation 2) Group 1 vs 3

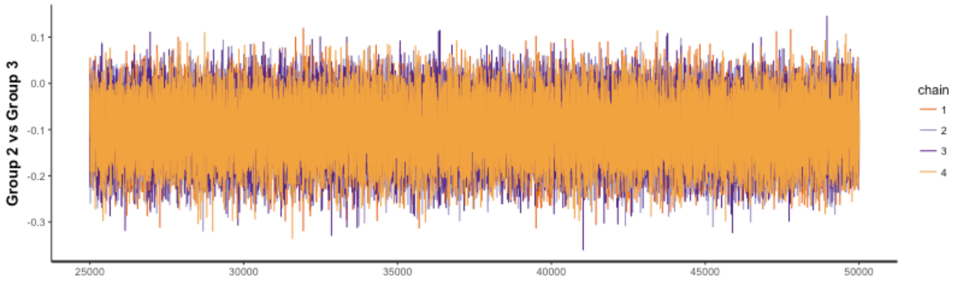

Figure 8 - Trace plot from Markov chain Monte Carlo sampling – risky drinking model (Equation 2) Group 2 vs 3
